# Antioxidant and Wound Healing Activity of Polyherbal Fractions of* Clinacanthus nutans* and* Elephantopus scaber*

**DOI:** 10.1155/2016/4685246

**Published:** 2016-07-26

**Authors:** Muhammad Shahzad Aslam, Muhammad Syarhabil Ahmad, Awang Soh Mamat, Muhammad Zamharir Ahmad, Faridah Salam

**Affiliations:** ^1^School of Bioprocess Engineering, Universiti Malaysia Perlis, Kompleks Pusat Pengajian Jejawi 3 (KPPJ3), Kawasan Perindustrian Jejawi, 02600 Arau, Perlis, Malaysia; ^2^Biotechnology and Nanotechnology Research Center, Malaysian Agricultural Research and Development Institute, 43400 Serdang, Selangor, Malaysia

## Abstract

*Elephantopus scaber* and* Clinacanthus nutans* are traditionally used as wound healing herb. The objective of the present study is to develop a new polyherbal formulation, by comparison, the herbal combination of* Elephantopus scaber* and* Clinacanthus nutans* as an in vitro antioxidant activity with their individual herbal activity followed by fractionation of polyherbal formulation for in vivo wound healing activities and identification of bioactive compounds from their active fractions. Antioxidant activity was performed in vitro by DPPH scavenging antioxidant activity followed by in vivo wound healing activities using excision wound model, incision wound model, and burn wound model. Toxicity of the fractions of the polyherbal formulation was performed by a dermal toxicity test. The result showed that* Elephantopus scaber* crude extract on the basis of EC_50_ performs a much faster action (15.67 *μ*g/mL) but with less % inhibition (87.66%) as compared to the combination of the new polyherbal formulation of crude extract (30 *μ*g/mL). The polyherbal formulation has the highest % inhibition (89.49%) at the same dose as compared to* Elephantopus scaber* (87.66%). In comparison among all crude and fractions of new polyherbal formulation, it was found that the ethyl acetate fraction of polyherbal formulation has the fastest activity (EC_50_ 14.83 *μ*g/mL) with % inhibition (89.28%). Furthermore, during evaluation of wound contraction on excision and incision wound model, ethyl acetate fraction possesses the highest activity with (*P* < 0.001) and (*P* < 0.0001), respectively. During burn wound model, aqueous fraction (*P* < 0.001) possesses the highest activity followed by an ethyl acetate fraction (*P* < 0.0001). LC-MS analysis discovered the presence of several flavonoid-based compounds that work synergistically with sesquiterpene lactone and other bioactive compounds. In conclusion, flavonoid increases the antioxidant activity that surges the rate of wound contraction and works synergistically with other bioactive compounds.

## 1. Introduction

Everyone experiences a fair share of wounds during the course of their lives. The impaired and aberrant wound healing imposes a huge financial burden in the developed world and is an insurmountable problem in the undeveloped one. Many new approaches, such as gene therapy and tissue engineered skin, have met with limited success. Conventional treatment of wound consists of nonsteroidal anti-inflammatory drugs (NSAIDs), antibiotics, and topical corticosteroid which have many drawbacks. All of them have some negative impact on healing [1, 2]. Herbal wound products have been an area of remarkable growth to understand the details of the wound healing response. The field of biologic wound products aims to accelerate healing by augmenting or modulating inflammatory mediators such as eicosanoids, cytokines, nitric oxide, and various growth factors [3]. Herb-herb combinations have been used in traditional Ayurvedic, Unani, Chinese, and Peruvian medicine practice for thousands of years, yet scientific evidence of potential bioactive compounds and their therapeutic benefits is lacking [4, 5].

The selection of herbs is based upon their activity and importance worldwide*. Clinacanthus nutans* Lindau is used for the treatment of burns, eczema, and herpes simplex. It has long been used as a traditional medicine for the treatment of insect and snakebites and skin rashes [6]. Payayor (*Clinacanthus nutans*) is a herbal therapy popular in Southeast Asia. A small study suggests that herbal Payayor may be superior to benzydamine for prevention of oral mucositis [7]. Leaves and even the whole plant of* Clinacanthus nutans* possess anti-inflammatory properties [8]. The plant contains lupeol, *β*-sitosterol, stigmasterol, botulin, and myricyl alcohol [9, 10]. It also contains six known C-glycosyl flavones isolated from n-BuOH and a water soluble portion of the methanolic extract of the stems and leaves such as vitexin, isovitexin, shaftoside, isomollupentin 7-O-*β*-glucopyranoside, orientin, and isoorientin. Five sulfur-containing glucosides were isolated from the n-butanol soluble portion of a methanolic extract of the stems and leaves of plant material [11]. A mixture of cerebrosides and monoacylmonogalactosylglycerol were separated from the leaves of* Clinacanthus nutans* [9].* Clinacanthus nutans* commonly known in Malaysia as Belalai Gajah identifies as areas of initial focus under Entry Point Project (EPP) by Government of Malaysia [12].

*Elephantopus scaber* is commonly known in Malaysia as Tutup Bumi. Hydroalcoholic extract possesses anti-inflammatory activity. A higher dose of compound significantly reduced carrageenan-induced pedal edema (57%) and formalin-induced pedal edema in rats (58%). Ethanolic extract of leaves has shown significant antiasthmatic activity, wound healing and nephroprotective activities along with its prominent antiplatelet activity. Deoxyelephantopin isolated from the ethanolic extract of leaves promotes significant wound healing activity by increasing cellular proliferation, the formation of granulation tissue, and synthesis of collagen and by increasing the rate of wound contraction [13]. Teng-Khia-U, a polyherbal formulation containing* Elephantopus scaber,* is a Taiwan traditional medicine formulated for treating nephritis, edema, dampness, chest pain, and fever/cough of pneumonia and scabies/arthralgia that was caused by the wound. Researchers have shown that Teng-Khia-U possessed hepatoprotective and anti-inflammatory activity [14, 15]. This research affords an opportunity to develop new herbal formulation using* Clinacanthus nutans* and* Elephantopus scaber* to determine the antioxidant profile in comparison with individual herbs. The active fractions of the polyherbal formulation will further evaluate for its in vivo wound healing activity and their bioactive compounds. The objective of the study was an investigation of the in vitro antioxidant activity of* Clinacanthus nutans*,* Elephantopus scaber*, and polyherbal extract of both medicinal herbs followed by their fractionation. After the antioxidant activity, fractions of polyherbal extract were further evaluated for their wound healing activities. Furthermore, the suggested idea of investigating bioactive compounds from active polyherbal fractions will help to identify several classes of phytochemicals such as flavonoids that are strongly associated with antioxidant and wound healing activity.

## 2. Material and Method

### 2.1. Plant Material

The leaf sample of these plant species was collected from the soil of Agrotech Research Centre, Institute Of Sustainable Agrotechnology, University Malaysia Perlis, Sg. Chuchuh Campus, Padang Besar, Perlis. The samples were brought to the laboratory and washed under running water to get rid of dirt. They were then dried under shade for two weeks. The materials were pulverized in an electric mixer and preserved in labeled glass bottles that were sealed and kept in the refrigerator for later use.

### 2.2. Preparation of Plant Extract

**Figure 1:**
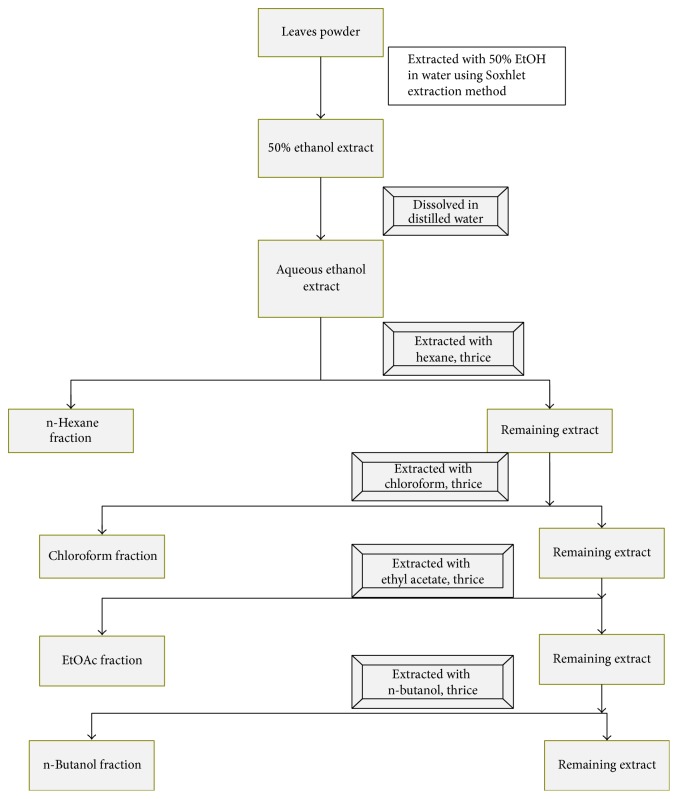
Extraction and fractionation of herb-herb combination of* Clinacanthus nutans* and* Elephantopus scaber.*

The conventional Soxhlet extraction apparatus has been used, consisting of a condenser, a Soxhlet chamber, and an extraction flask. The time period for Soxhlet extraction was 12 hours. 10 gm of dried and ground leaves of* Clinacanthus nutans, Elephantopus scaber,* and combination of both herbs in equal amount (1 : 1) was placed in a Soxhlet apparatus and extracted with 50% aqueous ethanol for 16 hours. The crude extract solutions obtained were then concentrated using a vacuum rotary evaporator (Eyela Rotavap N-1200, vacuum controller-NVC-2200, Eyela water bath OSB-2100, Eyela chiller CCA-1111 Rikakikai Co. Ltd., Tokyo) at a temperature of 50–60°C. The 50% ethanol extract was fractionated using different solvents, namely, hexane, chloroform, ethyl acetate, n-butanol, and water (Figure 1). The supernatant was filtered using Whatman number 1 sheet, pooled, and concentrated using vacuum rotary evaporator. The materials were dried in an oven at 37°C for 2 days. Dried powder was stored in screw-capped glass bottles and kept in a refrigerator at 4°C until further use.

### 2.3. Cream Formulation

20 gm of powder of* Clinacanthus nutans* and* Elephantopus scaber* was extracted by Soxhlet extraction. Filter it. 150 mL of a solution of the polyherbal formulation is mixed with equal amount of infused olive oil. After then we add 120 mg of honey, 100 mL of rest of solution of polyherbal extract, and 20 g of beeswax.

### 2.4. Free Radical Scavenging Activity Determination

The stable 2,2-diphenyl-1-picrylhydrazyl (DPPH) was used for determination of free radical scavenging activity of the extracts with few modifications [16]. Different concentrations (20–200 *μ*g/mL) of each crude herbal extract were added, at an equal volume, to an ethanolic solution of DPPH (0.1 mM). After 30 min at room temperature, the absorbance was recorded at 517 nm using UV/Visible Spectrophotometer (UV-1800 Shimadzu, Japan). The experiment was repeated for three times. Ascorbic acid was used as positive controls. The radical scavenging activity was expressed as the inhibition percentage and monitored as per the equation: % DPPH radical scavenging = (AC − AS/AC) × 100; AC = absorbance of control, and AS = absorbance of the sample solution. EC_50_ values denote the concentration of the sample, which is required to scavenge 50% of DPPH free radicals. It was calculated from inhibition curve.

### 2.5. Experimental Animals

Healthy, Swiss albino mice of either sex with more than 6–8 weeks of age and weight around 45–60 g were used. The animals were kept at room temperature (27 ± 2°C; 70–80% humidity; 12 h light/dark cycle) acclimatize for at least 72 h prior to the procedure. Commercial food pellets and water were supplied ad libitum. Animal handling and care were carried out throughout the experiment following international laboratory animal use and care guidelines [17].

#### 2.5.1. Grouping and Dosing of Animals

For excision model, six groups of mice, each containing four animals was used. The first group was treated with ethyl acetate fraction. The second and third groups were treated with n-butanol and aqueous fraction, respectively. The fourth group was treated with a polyherbal cream. The fifth and sixth groups were treated with control without drug and standard using povidone iodine, respectively. All administrations were performed topically, with a maximum quantity of 250 mg/kg/day. For burn wound model, five groups of mice, containing four mice per group, were used. The first group was treated with ethyl acetate fraction. The second and third groups were treated with n-butanol and aqueous fraction, respectively. The fourth and fifth groups were treated with standard using povidone iodine and control without drug, respectively. All administrations were performed topically, with a maximum quantity of 250 mg/kg/day. For incision wound model, all animals were treated in a similar fashion with excision wound model. For acute dermal toxicity, all animals were treated in a similar fashion with burn wound model.

### 2.6. Excision Wound Model

On a wounding day, animals were anesthetized using diethyl ether and the back hair of the animals was depilated by shaving. About 225 mm^2^ area was then marked and the full thickness of the marked area was carefully excised by using sharp sterilized scissors. After 24 h of wound creation, the sample was applied gently once daily, according to the respective grouping as described under grouping and dosing section, to cover the wounded area until complete healing was achieved. Wound contraction and epithelization period were monitored. Wound contraction was measured as millimeter (mm^2^) and percent contraction every day until complete epithelization was achieved [18].

#### 2.6.1. Measurement of Wound Contraction

The wound healing progress was evaluated by measuring wound areas using a transparency sheet and a permanent marker. The evaluated surface area was used to calculate the percentage of wound contraction, taking initial size of the wound (225 mm^2^) as 100% [19] as shown below:

(1) %  Wound  contraction=Initial  wound  size−specific  day  wound  sizeInitial  wound  size×100.

#### 2.6.2. Epithelization Time Measurement

The period of epithelization was calculated as the number of days required for falling off of the dead tissue remnants without any residual raw wound [20].

### 2.7. Incision Wound Model

**Figure 2:**
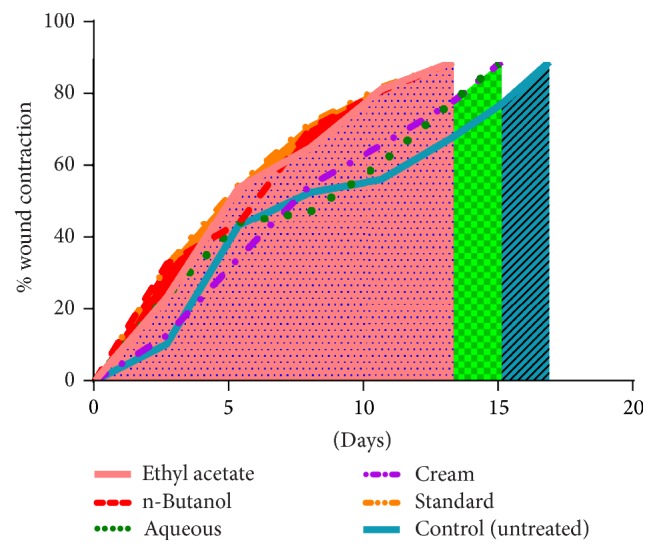
% wound contraction among all fractions as compared to control in excision wound model.

On a wounding day, animals were anesthetized in the same manner described for excision wound model. The dorsal fur of each mouse was then shaved and 3 cm long longitudinal paravertebral incision was made through the skin and subcutaneous tissue. The parted skin was then sutured 1 cm apart using a surgical thread and curved needle. The continuous thread on both wound edges was tightened for the good closure of the wounds (Figure 2). After 24 h of wound creation (on 1st day), animals were treated as described under grouping and dosing section, with a topical formulation of the vehicle, extract, or standard daily for nine days, leaving out the last group which did not receive any of the interventions. The sutures were removed on day 8 after incision and tensile strength was measured on the 11th postwounding day [21].

### 2.8. Burn Wound Model

The animals were anesthetized with diethyl ether and their dorsal surface was shaved with a sterile blade. The shaved area was disinfected with 70% (v/v) ethanol. Burn wounds were created on dorsal part of shaved rats using a metal rod (2.5 cm diameter) heated to 80–85°C and exposed for 20 s. After 24 h, dead tissues were excised using sterile surgical blade [22]. Measurement of wound contraction and epithelization time was calculated using excision wound model.

### 2.9. Acute Dermal Toxicity

For dermal toxicity, a total of 10 (5 females and 5 males) rats were used. Animals showing normal skin texture were housed individually in a cage and acclimatized to the laboratory condition for five days prior to the test. Following acclimation, around 10% of the body surface area fur was shaved 24 h before the study from the dorsal area of the trunk of the test animals. A limit test dose of 2000 mg/kg of each fraction was applied uniformly over the shaved area for 24 h. At the end of the exposure period, the residual test substance was removed and the animals were observed for development of any adverse skin reactions daily for 14 days [23].

### 2.10. LC-MS Analysis of Fractions

The chromatography was performed on an Agilent 1290 Infinity LC system coupled to Agilent 6520 Accurate-Mass Q-TOF mass spectrometer with dual ESI source using an Agilent Zorbax SB-C18 column (2.1 × 150 mm, 3.5 *μ*m). Flow rate was 0.5 mL/min and the injection volume was 1 *μ*L, column temperature 25°C, and autosampler temperature 4°C. Mobile phases consisted of 0.1% formic acid in water (A) and 0.1% formic acid in acetonitrile (B). The column was equilibrated for 5 min prior to each analysis. Separation was carried in 30 min under the following conditions: 0 min, 5% B; 5 min, 5% B; 20 min, 100% B; and 25 min, 100% B. The MS acquisition was performed in negative and positive ionization information acquisition (IDA) between* m/z* 100 and 1000, fragmentor voltage 125 V, skimmer 65 V, drying gas 10 L/min, gas temperature 300°C, nebulizer 45 psig, acquisition rate (spectra/s) 1.03, acquisition time (ms/spectrum) 973, and transients/spectrum 9632. The retention time and the mass obtained for the components were compared to Metabolomics database.

### 2.11. Chemicals

2,2-Diphenyl-1-picrylhydrazyl (DPPH radical) (Sigma-Aldrich Co., St. Louis, MO, USA), ethyl acetate, ethanol, toluene, chloroform, methanol (Fisher Scientific, UK), sulfuric acid, hydrochloric acid, ammonia, glacial acetic acid, ascorbic acid (HmbG), thin layer chromatography silica gel 60 F254 precoated plates, and formic acid (Merck, Darmstadt, Germany).

### 2.12. Statistical Analysis

Statistical analysis was performed using GraphPad Prism version 7 for Windows (GraphPad Software, San Diego, CA, USA) and Microsoft Excel 2013. Raw data obtained from different wound models are expressed as mean ± SEM. *P* values less than 0.05 were considered to be statistically significant. The data were analyzed using GraphPad Prism version 7 for Windows and differences among groups were compared by one-way ANOVA followed by Dunnett's test.

## 3. Results and Discussion

### 3.1. Free Radical Scavenging Activity Determination

All the results were reported based on the dry sample after Soxhlet extraction. Based on the results, the test conducted for evaluating antioxidant activity in* Clinacanthus nutans*,* Elephantopus scaber*, and a combination of an equal proportion of both leaves and their fraction (n-hexane, chloroform, ethyl acetate, n-butanol, aqueous fractions) showed that the DPPH radical scavenging activity increased with the increase in concentration.* Clinacanthus nutans*,* Elephantopus scaber,* and* new polyherbal combination* crude extract exhibited antioxidant activity at all the concentration of test solutions with maximum % inhibition by new polyherbal formulation (89.49%). With the increasing concentration of leaves extract (20–200 *μ*g/mL), the percentage of antioxidant activity also increased. On the basis of the highest % inhibition, the formulation was further fractionated into n-hexane, chloroform, ethyl acetate, n-butanol, and aqueous fractions. During evaluation of fractions of new polyherbal formulation, it was found that maximum % inhibition was achieved by an n-butanol fraction (89.97%) followed by ethyl acetate (89.28%) and aqueous fraction (86.20%), respectively. EC_50_ of all samples is mentioned in Table 1.

Data are expressed as the concentration necessary to scavenge 50% of DPPH radical. All measurements were repeated three times.

### 3.2. Excision Wound Model

**Figure 3:**
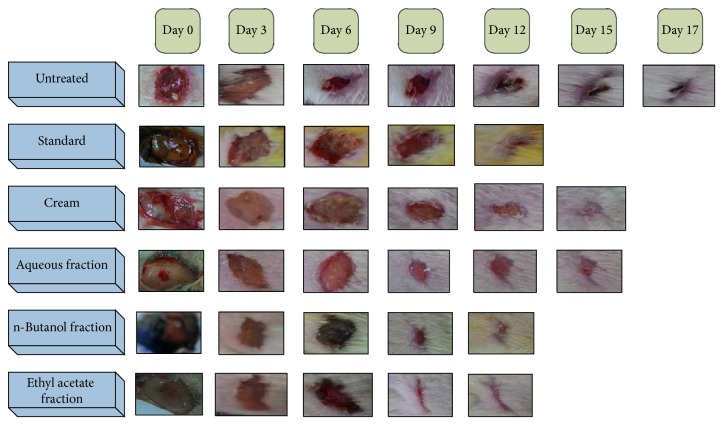
Comparison between all fractions with respect to control in excision wound model.

Significant wound contraction was initiated from day 6 in Group I (*P* < 0.05) and epithelization was completed on day 12 (*P* < 0.001), followed by Groups II (*P* < 0.001) and III (*P* < 0.0001) on day 12 and day 15, respectively (Table 2; Figure 3). Figure 2 represents % wound contraction among all fractions as compared to control in excision wound model.

### 3.3. Incision Wound Model

The tensile strength of animals treated with the ethyl acetate fraction was significantly higher (*P* < 0.0001) than the standard followed by n-butanol (*P* < 0.0001) and aqueous fraction (*P* < 0.0001) although no apparent difference was detected with cream of polyherbal extract (*P* < 0.001) as compared to n-butanol fraction (Table 3).

### 3.4. Burn Wound Model

Wound contraction is another parameter used to assess wound healing. Significant wound contraction was initiated from day 12 in Group III (*P* < 0.001), followed by Groups I (*P* < 0.0001) and II (*P* < 0.05) on day 15 (Table 4).

### 3.5. Acute Dermal Toxicity

In acute dermal toxicity studies, the rats of either sex were given ethyl acetate, n-butanol and aqueous fractions with doses (2000 mg/Kg/day body weight) for 10 days. The dose did not produce any signs of inflammation. The animals were physically active and were consuming food and water in a regular way. We did not notice any abnormal behavior.

### 3.6. LC-MS Profile of Active Fractions

**Figure 4:**
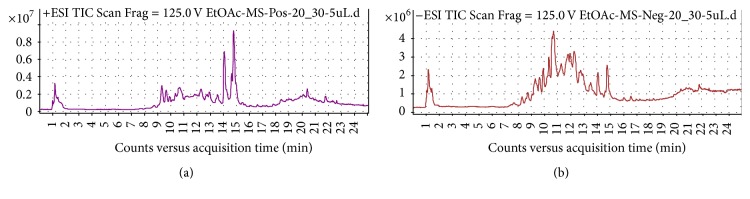
LC-MS report of (a) ethyl acetate MS^+^ and (b) ethyl acetate MS^−^.

**Figure 5:**
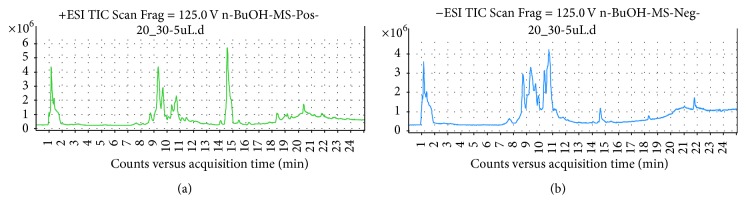
LC-MS report of (a) n-butanol fraction MS^+^ and (b) n-butanol fraction MS^−^.

**Figure 6:**
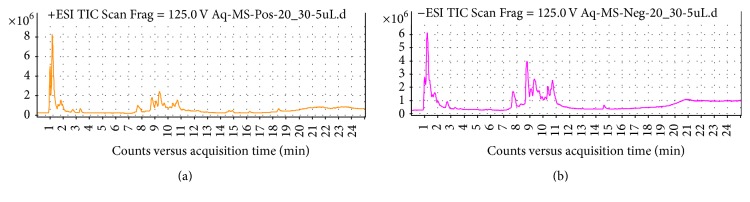
LC-MS report of (a) aqueous fraction MS^+^ and (b) aqueous fraction MS^−^.

**Figure 7:**
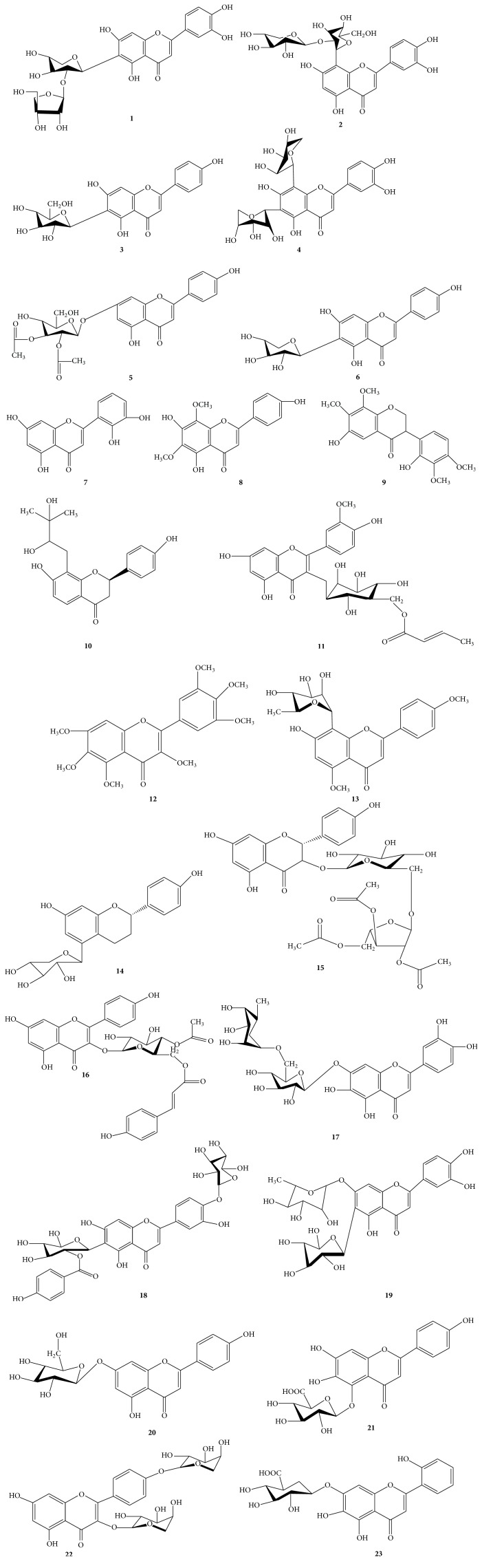
List of flavonoids identified from all fractions.

The molecular weight of the active compound was determined with electrospray ionization mass spectrometry (ESI-MS). The ESI-MS spectrum of the active compound is shown in spectrophotometer detection (LC-MS) provided by fragmentation pattern. Analysis can use ESI positive [M+H]^+^ ion and negative charges [M−H]^−^ ion. Figures 4, 5, and 6 represent the fragmentation pattern of the bioactive compounds detected from isolated ethyl acetate, n-butanol, and aqueous fraction, respectively. The LC-MS/MS results showed spectral data of possible flavonoids identified in all bioactive fractions (ethyl acetate, n-butanol fraction) of new polyherbal formulation from leaves of* Clinacanthus nutans* and* Elephantopus scaber* detected with MS in negative and positive modes. The spectral data from the peaks were identical and the identification was based on the LC-MS/MS data and comparison with the literature. The overview is shown in the chromatographic data of ethyl acetate, the n-butanol, and aqueous fraction which is summarized in Tables 5, 6, and 7, respectively. A maximum number of flavonoids were found in ethyl acetate fraction followed by an aqueous fraction and an n-butanol fraction. Flavonoids play an important role in wound healing [24–26]. There is another important bioactive compound also present in all fractions. Among them, sesquiterpene lactone and phenolic compounds are also present that showed the synergistic effect. Although no report was found on the synergistic effect of flavonoids and sesquiterpene lactone, further investigation is going on to understand the relationship between flavonoids and sesquiterpene lactone. List of possible flavonoids is also mentioned along with their structure in Figure 7.

## 4. Conclusion

Polyherbal formulation is a common practice in herbalism. An effort has been made to develop a new polyherbal formulation in the treatment of the wound. This is the first document that fractionates polyherbal extract and identifies the possible bioactive compound. This paper has established a correlation between wound healing activity and possible bioactive compounds. This paper indicates that flavonoids may perform an important role in the healing of the wound. A further experiment is required to isolate the possible bioactive compound from ethyl acetate and an aqueous fraction of polyherbal extract and to evaluate the isolated compounds on different wound model.

## Figures and Tables

**Table 1:** EC_50_ of crude *Clinacanthus nutans*, *Elephantopus scaber*, *Clinacanthus nutans + Elephantopus scaber* (polyherbal formulation), and their fractions.

Sample	EC_50_ (*µ*g/mL)
*Clinacanthus nutans*	103.28
*Elephantopus scaber*	15.67
*Clinacanthus nutans + Elephantopus scaber*	30.00
n-Hexane fraction	285.11
Chloroform fraction	248.22
Ethyl acetate fraction	14.83
n-Butanol fraction	89.74
Aqueous fraction	86.28
Ascorbic acid	8.20

**Table 2:** Mean area of wound (mm^2^) and % wound contraction during excision wound model.

Days	Day 0 (before treatment)	3	6	9	12	15	17
Group 1 (ethyl acetate)	229 ± 1.47 (0.00)	169.5 ± 2.10^*∗*^ (25.98)	90.25 ± 1.70^*∗*^ (60.58)	61.50 ± 1.84^*∗*^ (73.14)	19.50 ± 0.95^*∗∗*^ (91.48)	0.00 ± 0.00 (100)	0.00 ± 0.00 (100)
Group 2 (n-butanol)	231.25 ± 3.30 (0.00)	147 ± 1.22^*∗∗*^ (36.43)	117.5 ± 1.04 (49.18)	48 ± 1.22^*∗∗*^ (79.24)	21 ± 2.41^*∗∗∗*^ (90.91)	0.00 ± 0.00 (100)	0.00 ± 0.00 (100)
Group 3 (aqueous)	233.5 ± 5.89 (0.00)	171.5 ± 4.25^*∗*^ (26.55)	118 ± 3.36 (49.46)	109.5 ± 2.21^*∗∗*^ (53.10)	74.25 ± 1.93 (68.20)	28.50 ± 3.27^*∗∗∗*^ (87.79)	0.00 ± 0.00 (100)
Group 4 (cream)	228.25 ± 4.23 (0.00)	196 ± 2.94 (14.12)	140.75 ± 3.06^*∗∗*^ (38.33)	88 ± 1.22 (61.44)	59.5 ± 1.65^*∗∗*^ (73.93)	28.25 ± 1.18^*∗∗*^ (87.62)	0.00 ± 0.00 (100)
Group 5 (control)	229.25 ± 2.45 (0.00)	203.25 ± 2.49 (11.43)	118 ± 3.13 (48.52)	93.75 ± 3.75 (59.10)	84.5 ± 3.5 (63.14)	54 ± 4.08 (76.44)	30.5 ± 3.86 (86.69)
Group 6 (standard)	231.29 ± 4.73 (0.00)	148.25 ± 1.93^*∗∗*^ (35.89)	89.25 ± 2.28^*∗*^ (61.40)	44.25 ± 2.28^*∗*^ (80.86)	18.5 ± 2.72^*∗∗*^ (92)	0.00 ± 0.00 (100)	0.00 ± 0.00 (100)

Values are mean ± SEM (percent) of 4 rats in each group. ^*∗*^ *P* < 0.05, ^*∗∗*^ *P* < 0.001, and ^*∗∗∗*^ *P* < 0.0001 compared to respective day control group (statistical analysis was done by one-way analysis of variance followed by Dunnett's test for multiple comparisons).

**Table 3:** Breaking strength of ethyl acetate, n-butanol, and aqueous fraction of *Clinacanthus nutans* + *Elephantopus scaber* (polyherbal formulation).

Groups	Breaking strength (g)
Ethyl acetate	730.75 ± 7.56^*∗∗∗*^
n-Butanol	694.5 ± 6.34^*∗∗∗*^
Aqueous	624 ± 5.94^*∗∗∗*^
Cream	663.25 ± 5.37^*∗∗*^
Standard	408.75 ± 4.26^*∗∗*^
Control	337.5 ± 6.61

Values are mean ± SEM of 4 rats in each group. ^*∗∗*^ *P* < 0.001 and ^*∗∗∗*^ *P* < 0.0001 compared to respective day control group (statistical analysis was done by one-way analysis of variance followed by Dunnett's test for multiple comparisons).

**Table 4:** Mean area of wound (mm^2^) and % wound contraction during burn wound model.

Days	Day 0 (before treatment)	3	6	9	12	15	17	19
Group 1 (ethyl acetate)	499 ± 3.53 (0.00)	291.75 ± 3.44^*∗∗*^ (41.53)	264.5 ± 4.66^*∗∗*^ (46.99)	256.5 ± 2.84^*∗*^ (48.59)	60.5 ± 3.79^*∗∗*^ (87.87)	25.5 ± 2.53^*∗∗∗*^ (94.88)	0.00 ± 0.00 (0.00)	0.00 ± 0.00 (0.00)
Group 2 (n-butanol)	476.75 ± 9.25 (0.00)	371.75 ± 6.57^*∗*^ (22.02)	265 ± 5.49^*∗*^ (44.41)	200.75 ± 8.17^*∗∗*^ (57.89)	138.75 ± 7.4^*∗*^ (70.89)	59.75 ± 3.14^*∗*^ (87.46)	0.00 ± 0.00 (0.00)	0.00 ± 0.00 (0.00)
Group 3 (aqueous)	489.75 ± 1.84 (0.00)	311.25 ± 1.10^*∗∗*^ (36.44)	177.25 ± 2.75^*∗∗*^ (63.80)	81.5 ± 1.55^*∗∗∗*^ (83.35)	48.5 ± 1.55^*∗∗*^ (90.09)	0.00 ± 0.00 (0.00)	0.00 ± 0.00 (0.00)	0.00 ± 0.00 (0.00)
Group 4 (standard)	492 ± 4.02 (0.00)	318.5 ± 5.51^*∗∗*^ (35.26)	317.5 ± 3.37 (35.46)	255.5 ± 4.34^*∗∗*^ (48.06)	114.5 ± 3.77^*∗∗*^ (76.72)	28.25 ± 2.65^*∗∗*^ (94.25)	0.00 ± 0.00 (0.00)	0.00 ± 0.00 (0.00)
Group 5 (control)	496.5 ± 5.60 (0.00)	424.25 ± 8.40 (14.55)	324 ± 7.42 (34.74)	323.5 ± 7.5 (34.84)	207.25 ± 5.92 (58.25)	119.5 ± 6.13 (75.93)	56.5 ± 5.75 (88.62)	26.75 ± 5.50 (94.61)

Values are mean ± SEM (percent) of 4 rats in each group. ^*∗*^ *P* < 0.05, ^*∗∗*^ *P* < 0.001, and ^*∗∗∗*^ *P* < 0.0001 compared to respective day control group (statistical analysis was done by one-way analysis of variance followed by Dunnett's test for multiple comparisons).

**Table 5:** List of flavonoids tentatively identified in ethyl acetate fractions.

Characteristics	Ethyl acetate fraction MS^+^/MS^−^														
Sequence	1	2	3	4	5	6	7	8	9	10	11	12	13	14	15
*t*_*R*_	9.06	9.333	9.598	9.694	10.354	10.602	11.984	12.816	12.953	15.021	11.308	11.316	11.46	11.857	14.089
[M−H] (*m*/*z*)	580.1439	564.1492	448.1018	534.1379	516.1279	402.0963	286.0485	330.0746	362.1373	358.1422	546.1368	432.1426	446.1597	636.1493	390.1325
Error (ppm)	−1.91	−2.3	−2.7	−1.05	−2.16	−3.05	−2.49	−1.9	−1.95	−1.59	1.03	−1.31	−4.59	−2.26	−2.73
Molecular formula	C_26_H_28_O_15_	C_26_H_28_O_14_	C_21_H_20_O_11_	C_25_H_26_O_13_	C_25_H_24_O_12_	C_20_H_18_O_9_	C_15_H_10_O_6_	C_17_H_14_O_7_	C_19_H_22_O_7_	C_20_H_22_O_6_	C_26_H_26_O_13_	C_22_H_24_O_9_	C_23_H_26_O_9_	C_32_H_28_O_14_	C_20_H_22_O_8_
Proposed compound	A	B	C	D	E	F	G	H	I	J	K	L	M	N	O

A: isoorientin 2′′-O-apiofuranoside **1**; B: vitexin 2′′-O-xyloside **2**; C: scutellarein 6-glucoside **3**; D: 6,8-Di-C-beta-D-arabinopyranosylapigenin **4**; E: apigenin 7-(2′′,3′′-diacetylglucoside) **5**; F: cerarvensin **6**; G: 5,7,2′,3′-tetrahydroxyflavone **7**; H: 5,2′,4′-trihydroxy-6,8-dimethoxyflavone (rehderianin I) **8**; I: machaerol B **9**; J: brosimacutin B **10**; K: isorhamnetin 3-[6′′-(2-(E)-butenoyl)-glucoside] **11**; L: 3,5,6,7,3′,4′,5′-heptamethoxyflavone **12**; M: 5-hydroxy-7,8-dimethoxyflavanone 5-rhamnoside **13**; N: kaempferol 3-(4′′-acetyl-6′′-p-coumarylglucoside) **14**; O: apigeniflavan 5-O-xyloside **15**.

**Table 6:** List of flavonoids tentatively identified in n-butanol fractions.

Characteristics	n-Butanol fraction MS^+^/MS^−^						
Sequence	1	2	3	4	5	6	7
*t*_*R*_	9.058	9.468	9.699	10.363	8.735	8.928	9.328
[M−H] (*m*/*z*)	580.1439	448.101	534.1386	516.1279	706.1847	610.1533	564.1487
Error (ppm)	−1.86	−1.03	−2.37	−2.22	n.f	0.08	−1.35
Molecular formula	C_26_H_28_O_15_	C_21_H_20_O_11_	C_25_H_26_O_13_	C_25_H_24_O_12_	C_32_H_34_O_18_	C_27_H_30_O_16_	C_26_H_28_O_14_
Proposed compound	A	B	C	D	E	F	G

A: isoorientin 7-O-rhamnoside **19**; B: scutellarein 6-glucoside **3**; C = 6,8-Di-C-*β*-D-arabinopyranosylapigenin **4**; D: apigenin 7-(2′′,3′′-diacetylglucoside) **5**; E: kaempferol 3-[2′′′,3′′′,5′′′-triacetyl-*α*-L-arabinofuranosyl-(1 → 6)-glucoside **16**; F: 6-hydroxyluteolin 7-rutinoside **17**; G: vitexin 2′′-O-xyloside **2**; *t*_*R*_: retention time; n.f: not found.

**Table 7:** List of flavonoids tentatively identified in aqueous fractions.

Characteristics	Aqueous fraction MS^+^/MS^−^										
Sequence	1	2	3	4	5	6	7	8	9	10	11
*t*_*R*_	8.757	8.944	9.08	9.159	9.325	9.598	9.714	10.174	9.444	10.163	10.365
[M−H] (*m*/*z*)	730.1724	610.154	580.1445	564.1488	594.1595	448.1011	534.1385	462.0813	550.1329	462.0803	516.1266
Error (ppm)	2.96	−1.05	−2.81	−1.5	−1.74	−1.29	−2.08	−3.28	−1.15	−1.09	0.37
Molecular formula	C_34_H_34_O_18_	C_27_H_30_O_16_	C_26_H_28_O_15_	C_26_H_28_O_14_	C_27_H_30_O_15_	C_21_H_20_O_11_	C_25_H_26_O_13_	C_21_H_18_O_12_	C_25_H_26_O_14_	C_21_H_18_O_12_	C_25_H_24_O_12_
Proposed compound	A	B	C	D	E	F	G	H	I	J	K

A: isorientin 4′-O-glucoside 2′′-O-p-hydroxybenzoagte **18**; B: 6-hydroxyluteolin 7-rutinoside **17**; C: isoorientin 2′′-O-apiofuranoside **1**; D: vitexin 2′′-O-xyloside **2**; E: isoorientin 7-O-rhamnoside **19**; F: scutellarein 7-glucoside **20**; G: 6,8-Di-C-beta-D-arabinopyranosylapigenin **4**; H: scutellarein 5-glucuronide **21**; I: kaempferol 3,4′-dixyloside **22**; J: 5,6,7,2′-tetrahydroxyflavone 7-glucuronide **23**; K: apigenin 7-(2′′,3′′-diacetylglucoside) **5**.
